# *Haemonchus contortus* Adopt Isolate-Specific Life History Strategies to Optimize Fitness and Overcome Obstacles in Their Environment: Experimental Evidence

**DOI:** 10.3390/ani13111759

**Published:** 2023-05-25

**Authors:** Caroline Chylinski, Jacques Cortet, Jacques Cabaret, Alexandra Blanchard

**Affiliations:** 1 Archer Daniels Midland (ADM) International Sarl, A One Business Centre, La Pièce 3, 1180 Rolle, Switzerland; 2 ISP, INRAE, Université Tours, UMR1282, 37380 Nouzilly, France

**Keywords:** gastrointestinal nematode, establishment, fertility, fitness, egg-L3 larvae development, transcriptome

## Abstract

**Simple Summary:**

Ovine gastrointestinal nematodes (GIN) have flexible life history strategies, meaning they can alter the relative energy invested into each life trait in order to optimize their fitness under different environmental contexts. Some of the most prevalent ovine GIN species, such as *Haemonchus contortus*, have pervasive global distributions, and it is not known whether distinct isolates respond to the same environmental challenge with uniform alterations to their life history strategies or if different strategies affect different fitness outcomes. To investigate this further, this study compared the life history traits (i.e., establishment, fertility, egg-larvae development) and experimental fitness of three *H. contortus* isolates, following challenge in the parasitic (resistant vs. susceptible sheep) and free-living (summer, spring and winter climates) phases. The findings show *H. contortus* exhibit isolate-specific life history strategies to effectively maintain their fitness in spite of the environmental challenges. Partial exploration of the isolates’ transcriptomes further supports disparate expression profiles between them. These results bring new insights into the mechanisms by which GIN sustain their fitness across fluctuating environments, the results of which carry important implications for the sustainability of control interventions and in the potential comparability of experimental research.

**Abstract:**

Gastrointestinal nematodes (GIN) use flexible life history strategies to maintain their fitness under environmental challenges. Costs incurred by a challenge to one life trait can be recouped by increasing the expression of subsequent life traits throughout their life cycle. Anticipating how parasites respond to the challenge of control interventions is critical for the long-term sustainability of the practice and to further ensure that the parasites withstand favourable adaptive responses. There is currently limited information on whether distinct populations of a GIN species respond to the same environmental challenge in a consistent manner, with similar alterations to their life history strategies or comparable fitness outcomes. This study compared the life history traits and experimental fitness of three distinct *Haemonchus contortus* isolates exposed to environmental challenges at both the parasitic (i.e., passage through resistant or susceptible sheep) and free-living (i.e., exposure to diverse climatic conditions) life stages. The key findings show that *H. contortus* maintain their fitness under challenge with isolate-specific alterations to their life history strategies. Further, partial exploration of the *H. contortus* isolates transcriptomes using cDNA-AFLP methods confirmed disparate expression profiles between them. These results bring fresh insights into our understanding of the non-genetic adaptive processes of GIN that may hinder the efficacy of parasite control strategies.

## 1. Introduction

Over the last 50 years, considerable efforts have been invested into controlling gastrointestinal nematodes (GIN), yet they remain one of the leading threats to small ruminant production systems worldwide [1]. Anthelmintic drugs can effectively eliminate infections from the host. The mechanism by which this is achieved varies between the four main drug classes available [2]. Extensive overuse of these drugs, however, has rapidly been selected for resistance against them, including cases of multi-resistance against two or more drug classes [2]. The capacity of GINs to circumvent such control efforts is often attributed to their high underlying genetic diversity [3]. Mitigating the spread of anthelmintic resistance is crucial to preserving the efficacy of these drugs, which remains one of the most effective options in disease control. As such, there has been a shift in control efforts towards non-chemical control options to minimize the frequency in which anthelmintic treatments are required. These alternatives have a less direct impact on GIN populations [1] and mainly serve to interrupt the GIN life cycle, either by preventing contact with the free-living transmission stages (i.e., pasture management) [4] or by reducing their parasitic success in the host (i.e., selectively breeding the host for resistance) [5]. None of the alternatives offers absolute control over the GIN, which allows the surviving parasites the chance to respond to the challenge.

Even in the absence of human interference, GINs face greatly fluctuating environmental conditions throughout their life cycle, both when transmitting between hosts (i.e., climate, temporal and spatial variables) and surviving within the host (i.e., host protective immune responses) [6]. As low-mobility organisms, GIN cannot immediately escape habitats that become unfavourable, and a key survival strategy for such organisms is to alter their phenotype to adapt to their environment [7]. Many nematode species achieve this using flexible life history strategies to optimise their fitness (i.e., reproductive success, passing on their genes via offspring) under different environmental conditions [8]. Life history strategies refer to the expression of life traits which directly impact an organism’s survival and reproduction. In GIN, examples of life traits include the capacity of third-stage infective larvae (L3) to establish in the host; the arrested development of the fourth-stage larvae (L4) in response to unfavourable climatic environments; the growth of fifth-stage larvae (L5) into sexually reproductive adults; the production of viable eggs to develop into infective L3 offspring. The culminating success of these life traits results in their evolutionary fitness: a quantitative representation of how well an organism is able to survive and reproduce in its environment [9,10,11]. Life history theory conceptualises that key fitness components, such as growth, survival and reproduction, compete for resources from a finite energy budget, where energy directed into one component would be taken away from another [12,13]. In organisms with flexible life history strategies, these energetic investments are not fixed but can be re-distributed to optimise fitness in a given environment, as reflected by the relative expression of specific life traits.

Evidence of ovine GIN expressing flexible life history strategies has been observed previously in *Haemonchus contortus*, where L3 larvae exposed to desiccation stress prior to experimental infection showed an initial reduction in their establishment capacity compared to a control population; they consequently recouped the potential fitness losses with a significant increase to their reproductive output, ultimately maintaining comparable levels of fitness [14]. What remains unclear, however, is whether the adaptive response to environmental challenges is uniform across a GIN species or if distinct populations use different life history strategies to varying fitness effects. Ascertaining this information would have direct implications for the design of sustainable GIN control strategies that are capable of withstanding unfavourable adaptive outcomes. Several studies have already documented the differential expression of individual life traits when comparing GIN populations from different geographic origins [15,16], exposed to different husbandry conditions [17], with different anthelmintic resistance statuses [18,19,20,21] or maintained in different laboratory conditions [22,23,24]. It is not clear, however, whether these differences in individual life traits result in different fitness outcomes. This study aimed to determine whether *H. contortus* employs different life history strategies to navigate obstacles in their environment by comparing the life history traits and fitness of three distinct isolates under varying environmental challenges. Based on the differential expression of life traits observed between GIN isolates, we hypothesize they will exhibit isolate-specific life history strategies with disparate fitness outcomes across varying environments. The findings within this work highlight the continual biological processes that enable *H. contortus* to interact dynamically with their environment, overcome challenges and take advantage of opportunities in their life cycle.

## 2. Materials and Methods

### 2.1. Ethics Statement

All animal experiments were approved by the French Ministry of Teaching and Research and the regional Val de Loire Ethics Committee (CEEA VdL, no 19) as a protocol registered under the number 2012-06-10 in the experimental installations (n°agreement: C371753). All the protocols were conducted in accordance with EEC regulations (no. 2010/63/UE) governing the care and use of laboratory animals and have been effective in France since 1 January 2013.

### 2.2. Experimental Design

**Figure 1:**
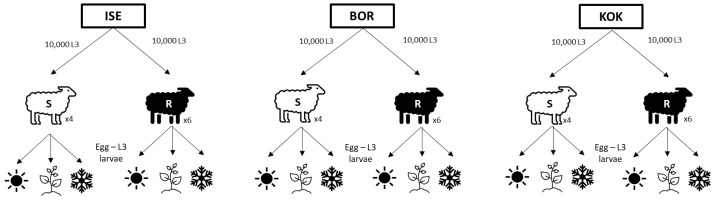
Experimental design. Each *H. contortus* isolate (ISE, BOR, KOK) was passaged through susceptible 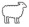 (×4) and resistant 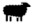 sheep (×6) using an infective dose of 10,000 L3 larvae; eggs excreted from the sheep were collected a cultured under conditions reflecting summer 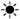, spring 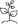 and winter 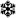.

The study was designed to compare the life history strategies and fitness of three *H. contortus* isolates under different environmental conditions, including passage of the isolates through two host conditions (i.e., susceptible and resistant to GIN infection) and culturing the free-living stages under three climatic conditions (i.e., reflecting spring, summer and winter) (Figure 1).

### 2.3. Haemonchus Contortus Isolates

Three different *H. contortus* isolates were selected for comparison (Table 1): (i) ISE: an isolate susceptible to all classes of anthelmintics [25]; (ii) RHS6 *aka* BOR: an isolate that had been selected experimentally for high levels of resistance against levamisole [26]; (iii) KOK: an isolate which is partly resistant against the three main anthelmintic classes (unpublished data), i.e., levamisole, avermectin, benzimidazole, originally obtained from a farm in South Africa and kindly donated by J. Van Wyk in 2000. None of the isolates had been experimentally exposed to resistant sheep prior to the study.

### 2.4. Sheep

Twelve Berrichon du Cher lambs (4 months old) and 18 Martinik Blackbelly rams (18 months old) were used in the study, both of which originated from populations bred at INRAE in Bourges, France. The Martinik Blackbelly breed had been selected based on its resistant [27] phenotype against *H. contortus* infection. The age differences between the sheep were selected to accentuate the relative protective responses to GIN, where younger sheep are considered to be more susceptible to infections [28]. Prior to the study, all sheep were parasite naïve and maintained indoors in GIN-free conditions, confirmed by several negative faecal egg counts (FEC). All sheep were fed hay and feed concentration *ad libitum* and had open access to water.

### 2.5. Haemonchus Contortus Experiment

Each *H. contortus* isolate was passaged through four susceptible lambs and six resistant rams, administered *per os* with a single infective dose of 10,000 L3 larvae. The infection lasted 61 days, after which the sheep were slaughtered and necropsied.

### 2.6. Pathogenicity

Pathogenicity was calculated based on the total reduction in haematocrit from day 0 to day 53 post-infection (dpi), divided by the total number of worms counted at necropsy per individual sheep. Blood samples were drawn from the sheep’s jugular vein into a dry tube using a 10 mL gauge syringe. Haematocrit percentage was determined using a manual Hawksley micro-haematocrit reader.

### 2.7. Faecal Nematode Egg Counts (FEC)

Faecal samples were collected from all sheep on 0, 19, 21, 27, 29, 30, 34, 40, 42, 58 and 61 dpi to carry out FEC using a modified McMaster technique [29] in a sodium chloride flotation solution accurate to 50 eggs per gram (EPG) of faeces. These days were selected to capture the changing dynamics in FEC observed throughout *H. contortus* infections, including the pre-patent period at approximately 21 dpi and the peak in egg output around weeks 4–5 post-infection [30].

### 2.8. Egg-L3 Larvae Development

Faeces collected from sheep on 27, 29 and 30 dpi were used to measure the capacity of *H. contortus* eggs to develop into infective L3. This is a window when *H. contortus* FEC peaks [30], and the isolate was therefore considered to have high fitness potential. This was carried out on all four susceptible Berrichon du Cher lambs, but due to the labour-intensive nature of the technique, it was done on only three of the six resistant Martinik Blackbelly rams from each treatment group. The three rams with the highest FEC on 27 dpi were selected and sampled at each time point.

Egg-L3 development assays were carried out by culturing 3 × 3 g faecal samples for each sheep, under three different climatic conditions: (i) spring: optimal conditions for *H. contortus* development [31], i.e., 23 °C, 70% Humidity (H), 10 days (ii) summer (heat stress): i.e., 28 °C, 80% H, 5 days; (iii) winter (cold stress), i.e., 18 °C, 60% H, 15 days.

The L3 were extracted from the faecal matter using the Baermann funnel technique over a 24 h period at room temperature [32] and counted under a microscope to obtain the number of L3 developed per 3 g of faeces. Only living L3 were included in the count. The number of L3 counted for each 3 g sample was divided by three to obtain the number of L3 per 1 g of faeces, which was divided by the FEC to obtain the proportion of eggs that develop into L3 per gram of faeces. To evaluate the relative impact of passage through resistant or susceptible sheep on *H. contortus* isolate egg-L3 larvae development and fitness in the absence of climatic stress, calculations were based on results obtained from spring conditions.

### 2.9. Establishment

The establishment capacity of the L3 larvae was based on the number of adult worms counted in the host abomasum at necropsy (61 dpi), based on procedures detailed in [33] and expressed as a percentage of the infective dose. Although the presence of L4 larvae was not expected as late as 61 dpi, the abomasal mucosa was incubated in water at 37 °C for four hours, washed thoroughly in water, which was sieved through a 30 µm mesh to collect and count any remaining larvae.

### 2.10. Fertility

The fertility measure reflected the number of eggs per individual female and was calculated based on procedures outlined by [34]. In brief, the total daily egg output per sheep was determined by multiplying the FEC on 61 dpi by the total quantity of faecal matter (QFM) produced in a day. The QFM (kg) was calculated using the following formula (back-transformed from logarithm) developed specifically to the conditions of this study, based on a linear regression between the logarithm of the weight of faecal excretion collected over a 24 h period and the logarithm of the metabolic weight (W^0.75^) of Berrichon du Cher lambs and Martinik Blackbelly rams [35]:

QFM = 0.041W^0.75^ p = 0.00; r = 0.95, (1)

where W is the weight of the individual sheep (kg).

The total daily egg output (FEC × QFM) was then divided by the number of adult female worms found at necropsy to determine the fertility.

### 2.11. Parasite Fitness

The experimental fitness of the *H. contortus* isolates was calculated by dividing the number of L3 larvae produced in the second generation (F2) by the initial infective dose (F1). Specifically, the number of L3 larvae observed in the development assays (L3 per 1 g faeces) was multiplied by the QFM to provide the total number of living L3 produced by an *H. contortus* by an individual sheep per day. This was then divided by the number of L3 in the infective dose (i.e., 10,000) to provide the experimental fitness value. Calculations of experimental fitness were based on samples taken at 27, 29 and 30 dpi, the same time points used for the egg-L3 larvae development assays.

### 2.12. Statistical Analyses

Inter-isolate differences in the life traits and fitness measures were compared across each of the environmental conditions, and their relative patterns of phenotypic expression were presented on radar graphs. Intra-isolate comparisons were also carried out to identify variations in the isolate’s performance across different environmental conditions. Statistical analyses were based on a general linear model (GLM) with SPSS software Version 11.5. Factors included the breed of sheep, isolate and climatic conditions. ANOVA was also performed on each of these factors. The FEC and fitness variables were log-transformed prior to analyses to normalize variance.

### 2.13. RNA Extraction and Double Strand cDNA Synthesis on H. contortus Adult Males

RNA extractions for cDNA-AFLP analyses were carried out on all three *H. contortus* isolates following infection on resistant sheep. For each isolate, the total RNA from a pool of 10 adult males, stored at −80 °C in RNAlater, was extracted as per the manufacturer’s instructions following homogenization in Trizol reagent (Life Technologies). RNA pellets were dissolved in 50 µL of RNAse-free water and stored at −80 °C. Each sample was treated with DNase I (Promega, Madison, WI, USA), according to the manufacturer’s instructions, for 30 min at 37 °C to avoid genomic DNA contaminations.

The synthesis of the first strand cDNA was performed in a mix containing 5 µg of total RNA, 5 µM of d(T)25 primer, 0.5 mM of each dNTP and a sufficient quantity of RNAse-free water to complete at 13 µL. Each sample was heated for 10 min at 70 °C and set on ice. A 1× superscript buffer, 40 U of RNasin (Promega), 5 µM of dithiothreitol (DTT) and 200 U of superscript II (Invitrogen) were then added. The mix was incubated at 42 °C for 1 h, then at 70 °C for 15 min and stored at −20 °C until use.

The second cDNA strand was synthesized in a mix containing 20 µL of the first strand cDNA, 1× second strand buffer (NEB2 buffer), 0.2 mM of each dNTP, 20 µM of DTT, 40 U of *E. coli* DNA Polymerase I (NEB), 15U of DNA ligase (NEB), 6 U of RNAse H (NEB) in 150 µL of sterile distilled water. The mix was incubated at 16 °C for 2 h. The double-stranded cDNA was then purified using phenol/chloroform/isoamyl alcohol (25:24:1) and resuspended in 40 µL of sterile distilled water.

### 2.14. cDNA-AFLP Experiments

The cDNA samples were digested with HindIII/MseI (NEB), restriction fragments were ligated with their corresponding adap2 G_7/ters and pre-amplification was carried out during 30 cycles (94 °C 30 s, 55 °C 45 s, 72 °C 60 s) using primers without selective nucleotide H + 0 and M + 0 (Table 2). PCR was then performed with selective primers (detailed below) and a trace amount of [γ^33^P]-labelled 5′ primer using the following program: 94 °C for 2 min, 14 cycles (94 °C for 30 s; 60 °C–56 °C for 30 s (the temperature decreasing slightly after each cycle to reach 56 °C by the 14th cycle) 72 °C for 1 min), 24 cycles (94 °C for 30 s, 56 °C for 30 s, 72 °C for 1 min), 72 °C for 5 min. The amplification products were separated on a 5% polyacrylamide gel and analyzed after exposure to X-ray film for 24–48 h. Several combinations of primers were used for cDNA-AFLP, but only 16 were selected for consequent analyses on the basis that they provided the clearest band profiles. These included H + A + M12, M14, M15, M16, M17, M18 M19, M20, M24, M26; H + T + M15, M16, M19; H + T + M16, M23, M25. Details of these primers can be viewed in Table 2.

### 2.15. Transcriptomic Expression Profiles

The amplified cDNA fragments appear as band profiles on the X-ray film, and each individual band is referred to as a transcript-derived fragment (TDF). Polymorphisms in transcriptomic expression are evident by the relative presence or absence of TDFs across the *H. contortus* isolates. In total, 689 TDFs were compared across the isolates. In this study, we did not attempt to associate the TDFs to specific *H. contortus* life traits. Instead, the intent was to conduct a preliminary exploration to ascertain whether such a detailed approach would be warranted based on the divergence of the isolate’s transcriptomes. The closer the isolates are positioned on the results graph, the greater their transcriptomic similarity. The inertia of each axis corresponds to the part of variance explained by the axis. Each axis corresponds to a synthetic variable which results from a combination of variables in such a way that it maximises the inertia. Each axis is independent of the others and thus brings its own information. Since the number of bands was important, the explanatory value of each of the three axes was high, and they were significant [36].

## 3. Results

### 3.1. Inter-Isolate Comparison of Life History Strategies and Fitness in Susceptible Lambs × Spring Conditions

**Figure 2:**
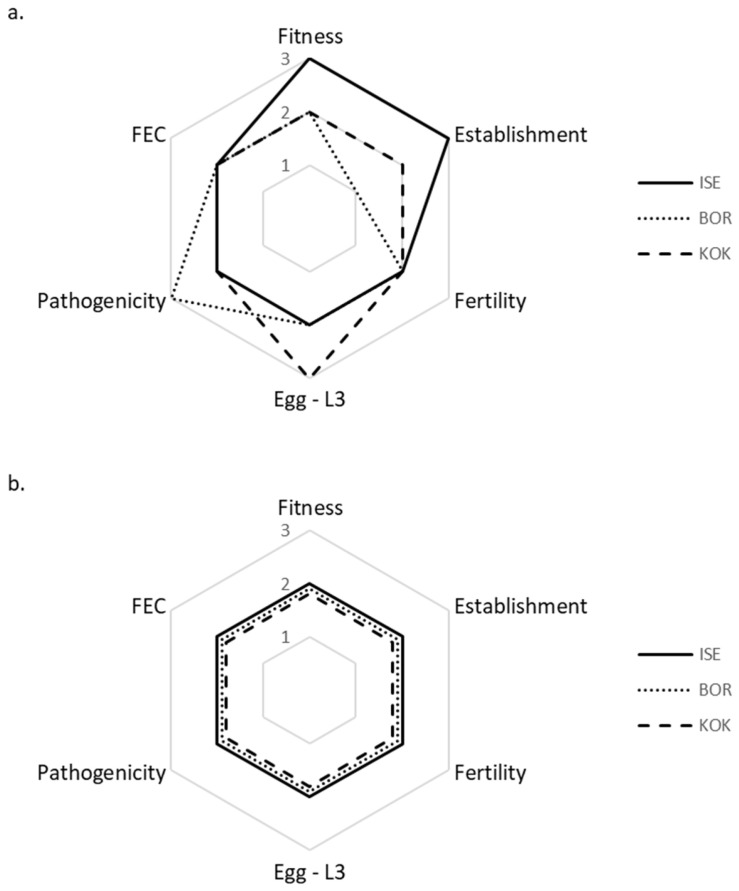
Comparison of life trait expression and fitness of three *H. contortus* isolates, ISE (solid line), BOR (dotted line) and KOK (dashed line) following infection in susceptible (**a**) and resistant (**b**) sheep. Free-living stages were cultured under optimal spring climatic conditions. The axes represent significant differences between the *H. contortus* isolates (ANOVA *p* ≤ 0.05): 1 = significantly reduced; 2 = no significant difference; 3 = significantly increased life trait expression or fitness.

Following infection in susceptible sheep, the three *H. contortus* isolates differed significantly in their expression of life history traits and fitness (Figure 2a). Each isolate outperformed the others in at least one of the traits measured: ISE had a significantly greater establishment capacity than BOR or KOK; BOR was significantly more pathogenic than ISE or KOK; KOK had a significantly greater egg-L3 development ratio than ISE or BOR. Under these conditions, the life history strategy of ISE resulted in significantly greater fitness than BOR or KOK.

### 3.2. Inter-Isolate Comparison of Life History Strategies and Fitness in Resistant Rams × Spring Conditions

Following infection in resistant sheep, there were no significant differences in the expression of life traits or fitness between the three *H. contortus* isolates (Figure 2b).

### 3.3. Intra-Isolate Comparison of Life History Strategies and Fitness in Susceptible vs. Resistant Sheep × Spring Conditions

**Figure 3:**
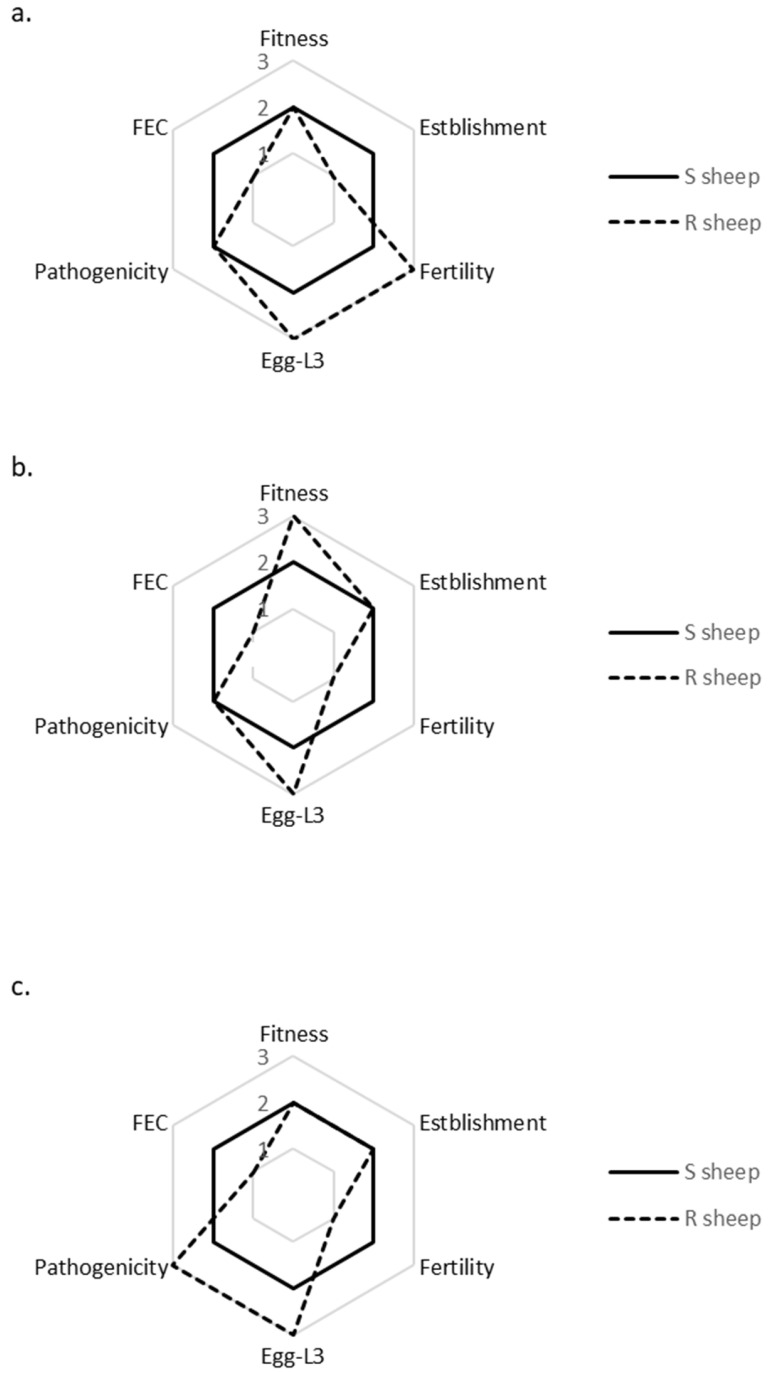
Comparison of life trait expression and fitness of three *H. contortus* isolates: ISE (**a**), BOR (**b**) and KOK (**c**) following infection in susceptible (solid line) and resistant sheep (dotted line). Free-living stages were cultured under optimal spring climatic conditions. The axes represent significant differences within each of the *H. contortus* isolates (ANOVA *p* ≤ 0.05), where life trait expression and fitness in susceptible sheep was normalized onto axes 2 = no significant differences; axes 1 = significantly reduced; axes 3 = significantly increased, life history trait expression or fitness in resistant sheep.

Passage through resistant sheep significantly altered the expression of life traits in all isolates compared to their performance in susceptible sheep (Figure 3a–c; Table 3). Changes to life history traits were isolate-specific. ISE incurred a significant, nine-fold reduction in establishment capacity (Figure 3a). The fertility of BOR (Figure 3b) and KOK (Figure 3c) significantly decreased by 19-fold and five-fold, respectively, in resistant sheep, whereas that of ISE (Figure 3a) significantly increased five-fold. All isolates significantly augmented their egg-L3 development following passage in resistant sheep seven-fold, eight-fold and six-fold in ISE, BOR, and KOK, respectively (Figure 3a–c). The pathogenicity of KOK significantly doubled in resistant sheep (Figure 3c). Ultimately, the isolates maintained (ISE, KOK), or increased (BOR), their fitness levels in resistant sheep (Figure 3a–c).

### 3.4. Isolate Egg-L3 Development under Different Climatic Conditions

**Figure 4:**
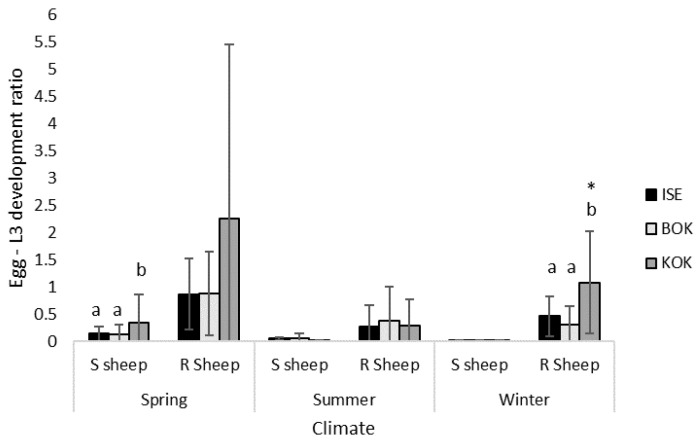
Mean egg-L3 larvae development ratios (±SEM) for three *H. contortus* isolates, ISE, BOR and KOK, following passage in either susceptible (S) or resistant (R) sheep and culture under either spring (23 °C, 70% H, 10 days), summer (28 °C, 80% H, 5 days) or winter (18 °C, 60% H, 15 days) conditions. Significant inter-isolate differences are denoted with different letters (ANOVA *p* ≤ 0.05). Significant intra-isolate differences, where fitness is greater following infection in resistant sheep compared to susceptible sheep, denoted with * (ANOVA *p* ≤ 0.05).

Following infection in susceptible sheep, KOK expressed a significantly greater egg-L3 development ratio than ISE or BOR under spring conditions (Figure 4; Table 3). There is a general trend in the data showing the egg-L3 development under all climatic conditions was greater following infection in resistant sheep, although this interaction was only statistically significant for KOK under winter conditions, showing a 101-fold increase. Technically, results for the egg-L3 development ratio should not exceed 1, which would reflect that 100% of the eggs were developing into L3 larvae. The results here likely reflect inaccuracies in some of the measures used to calculate this, such as FEC.

### 3.5. Isolate Experimental Fitness under Different Climatic Conditions

**Figure 5:**
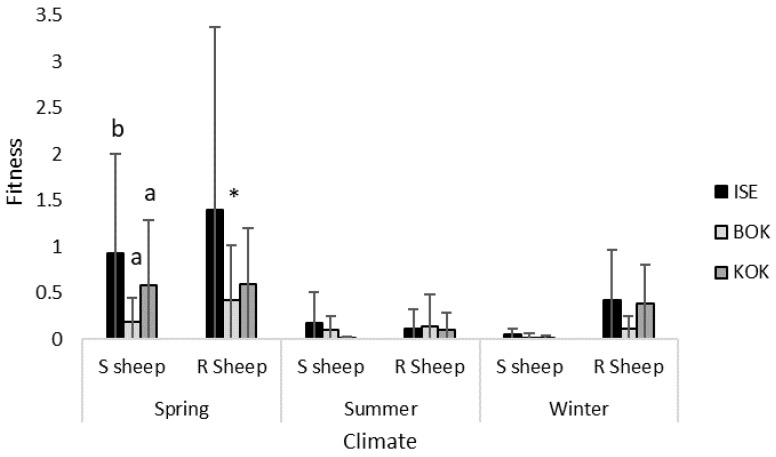
Mean fitness (±SEM) for three *H. contortus* isolates, ISE, BOR and KOK, following passage in either susceptible (S) or resistant (R) sheep and culture under either spring (23 °C, 70% H, 10 days), summer (28 °C, 80% H, 5 days) or winter (18 °C, 60% H, 15 days) conditions. Significant inter-isolate differences are denoted with different letters (ANOVA *p* ≤ 0.05). Significant intra-isolate differences, where fitness was greater following infection in resistant sheep compared to susceptible sheep, denoted with * (ANOVA *p* ≤ 0.05).

Significant differences in fitness were only observed between the isolates following passage in susceptible sheep and culture under spring conditions (Figure 5; Table 3). BOR had a significantly greater fitness following passage in resistant sheep compared to susceptible sheep.

### 3.6. Genetic Analyses of the H. contortus Isolates

**Figure 6:**
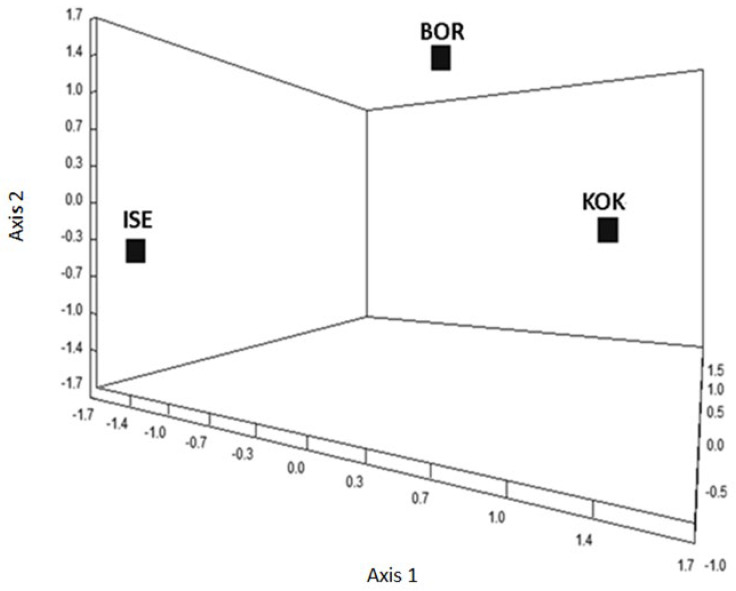
Correspondence analysis representing the genetic proximity of the three *H. contortus* isolates, ISE, BOR and KOK, based on AFLP transcriptomic analyses. The three-dimensional axes reflect a linear combination of all variables examined (i.e., TDFs).

Partial exploration of the isolate’s transcriptome identified a combined total of 689 TDFs. Of these, ISE had 401, BOR 309 and KOK 459. Approximately one-third of the TDFs (211) identified were expressed in all three isolates. Correspondence analyses comparing the patterns of transcriptomic expression between the isolates showed the isolates to be significantly distinct genetically (Figure 6). The distances between isolates indicate their genetic distances. The three-dimensional axes reflect a linear combination of all variables examined (i.e., TDFs) representing a similar value of inertia (37, 35 and 28% of the total variance).

## 4. Discussion

The principal outcome of this study shows *H. contortus* to exhibit isolate-specific life history strategies, which exhibit phenotypic plasticity to maintain their fitness in response to environmental challenges. These results bring fresh insights into our understanding of the adaptive processes which may be affecting alternative control approaches.

The distinct isolate-specific life history strategies were apparent on the background of susceptible sheep, where each isolate surpassed the others in the expression of at least one life trait: ISE had a superior establishment capacity, KOK invested greater resources into the egg-L3 development and BOR was the most pathogenic. The significantly greater level of fitness obtained by ISE indicates that in susceptible lambs, their life history strategy was most successful. The greater establishment capacity of ISE was, at least in part, a contributing factor, although it is also likely that other phenotypic traits, which may not be amenable to measurement, also impacted the fitness outcome [37]. Under different environmental conditions, one of the other isolates may have had a life history strategy that resulted in higher fitness outcomes.

Several interesting observations emerged following infection in resistant sheep. Firstly, this host environment diminished any significant differences between the isolate’s life trait expression or fitness, suggesting none of them had a life history strategy that conferred a competitive fitness advantage. Similar findings have been recorded previously. In a study comparing two *H. contortus* isolates from distinct climatic regions, the isolates exhibited differences in the expressions of certain life traits, including length of the pre-patent period and the rate of larval arrested development, when administered as fresh larvae, yet these differences disappeared when the isolates were challenged with cold storage prior to infection [16].

Secondly, passage through resistant sheep impacted the *H. contortus* isolates differently, where ISE incurred a significant reduction in establishment compared to passage in susceptible sheep, BOR and KOK incurred a significant reduction in fertility. The Martinik Blackbelly was used as the resistant breed of sheep in this study, having been selected over time for its lower FEC and increased resilience to GIN [38]. Previous studies have demonstrated the breed to negate both establishment and fertility in a single *H. contortus* isolate [27]. To our knowledge, this is the first study to observe disparate regulatory effects of resistant sheep across different GIN isolates. Thirdly, all of the isolates responded to the challenge of resistant sheep with dissimilar facultative changes to their life trait expression when compared to their performance in susceptible sheep: ISE increased both fertility and egg-L3 development; BOR only increased their egg-L3 development; KOK increased egg-L3 development and their pathogenicity. As a result, all three isolates either maintained (ISE, KOK) comparable levels of fitness in resistant sheep to what they attained in susceptible sheep, or in the case of BOR, it even improved.

There were certain limitations in the measurements used to capture the life traits of *H. contortus* in this study. The technique used to measure FEC, for example, underestimated the number of eggs present. This explains why in the egg-L3 development results, more larvae were counted than eggs to hatch them. As FEC is a ratio (eggs per gram of faeces), any factor which alters the volume and/or consistency of faeces, such as the dry matter in feed and/or diarrhoea, can impact the reliability of the measure [39,40]. Studies have also shown FEC to be most reliable when applied to composite faecal samples as an indicator of the group mean [41] as opposed to individual sheep, as used in this trial. Rossangio and Gruner [42] developed a different method for FEC which was shown to be correlated to the technique used in this trial but ultimately gave higher values. This method, however, is more labour intensive and was not practical for use in this trial. Likewise, alternative methods exist to determine GIN fertility based on measuring the length of the female worms as an indicator of the number of eggs in utero [43]. However, the presence of eggs does not necessarily mean these eggs will be released, a factor which is captured by the FEC, and it also requires the sheep host to be necropsied to recover the worms, which clearly interrupts obtaining data on the dynamics of fertility over time that FEC can monitor. The climatic stresses applied to the free-living egg-L3 larvae stages in the study did not reveal any isolate-specific effects. Although not deemed significant, there was a notable trend in the data whereby egg-L3 development and, to a lesser extent, fitness under all climatic conditions were enhanced in the isolates following infection in resistant sheep. Studies in the free-living nematode *Caenorhabditis elegans* documented that offspring originating from harsher maternal environments tended to be better primed to survive poor conditions thereafter [44]. Exposing GIN to control solutions that confer a fitness advantage for successive generations would clearly be a counterproductive outcome for control, and developing approaches that can withstand unfavourable parasitic evolution is integral to their sustainability. Further, recent experimental findings in the ovine GIN *Teladorsagia circumcincta* observed that serial passage in resistant sheep selected offspring with increased infectivity and fitness in just three generations [35].

This study intentionally selected *H. contortus* isolates with highly diverse backgrounds on the basis of heterogeneities in their geographic origin, anthelmintic resistance status and laboratory treatment could potentially shape different life history strategies through local adaptation. While this was confirmed in the study, their varied backgrounds preclude the possibility of correlating the observed fitness outcomes to specific factors, such as their anthelmintic resistance status. Answering this would be very challenging and require that isolates were artificially selected for specific differences (i.e., anthelmintic resistance) from the same founding population over a sufficient number of generations to effect genetic adaptations while simultaneously controlling for the impact differential laboratory treatments can have on the life history strategies and fitness of GIN [23]. Partial exploration of the three *H. contortus* isolates transcriptomes following infection in resistant sheep confirmed disparate expression profiles between them. From the total identified TDFs, the isolates expressed less than a third in common. *Haemonchus contortus* is well documented to maintain high levels of genetic diversity, most of which occurs within regional populations as opposed to between them [45]. Rather than providing an exhaustive list of differentially regulated genes between the isolates, this work intended to gauge the potential merits of correlating transcriptomic expression profiles to the phenotypic success of the isolates, an approach that has proven useful in the identification of candidate genes involved in anthelmintic resistance [46,47,48,49]. With a more complete analysis of the isolate’s transcriptome, combined with quantitative RNA-sequencing techniques, it may be possible to correlate differentially expressed genes to differentially expressed life traits, such as establishment, to identify the key genes involved in components of *H. contortus* fitness, and warrants further study.

## 5. Conclusions

Recognising GIN as parasites capable of responding in real-time to obstacles in their life cycle is critical to developing sustainable control strategies. We cannot expect all *H. contortus* isolates to respond to challenge in a uniform manner, and it carries important implications for experimental research, in the comparability of studies using different isolates, and for the value of control tools, which will vary across different farms, framing different adaptive responses. Based on our findings, we propose that the long-term viability of control efforts could benefit from targeting GIN fitness rather than focusing on individual life traits, such as reducing FEC. The measure used in the present study to quantify fitness is highly amenable to experimental work. It is worth noting that at no point in this study did FEC serve as an accurate reflection of fitness, and it is not recommended to rely on this indicator for such purposes. It is not possible to measure fitness in farm conditions, however, and our findings support previous calls for an integrated approach to parasite management [50,51] where different controls tools are employed to exert pressure on several parasite stages (i.e., adult worms and the presence of infective larvae) simultaneously, thus limiting the prospects for adaptive responses.

## Figures and Tables

**Table 1:** The origin of the three *H. contortus* isolates compared in the study.

Isolate	Country of Origin	Years Maintained in Lab (INRAE)	Anthelmintic Resistance Status	Selection of Anthelmintic Resistance	Prior Experience of Resistant Sheep	Line History Reference
ISE	NA (inbred isolate)	25+	Susceptible	NA	No	[26]
BOR	Netherlands	10	Levamisole	Experimentally	No	[25]
KOK	South Africa	18	Levamisole, Avermectin, Benzimidazole	On field	No	Donated by J. Van Wyk, 2000

Not applicable (NA).

**Table 2:** Primer sequences used in cDNA-AFLP analyses.

Primer Name	5′-3′
M + 0	GATGAGTCCTGAGTAA
H + 0	GACTGCGTACCAGCTT
H + A	GACTGCGTACCAGCTTA
H + T	GACTGCGTACCAGCTTT
M12	GATGAGTCCTGAGTAAAC
M14	GATGAGTCCTGAGTAAAT
M15	GATGAGTCCTGAGTAACA
M16	GATGAGTCCTGAGTAACC
M17	GATGAGTCCTGAGTAACG
M18	GATGAGTCCTGAGTAACT
M19	GATGAGTCCTGAGTAAGA
M20	GATGAGTCCTGAGTAAGC
M23	GATGAGTCCTGAGTAATA
M24	GATGAGTCCTGAGTAATC
M25	GATGAGTCCTGAGTAATG
M26	GATGAGTCCTGAGTAATT

**Table 3:** Mean (±SEM) performance of *H. contortus* isolate life traits and fitness following infection in susceptible and resistant sheep.

	Susceptible Sheep			Resistant Sheep		
	ISE	BOR	KOK	ISE	BOR	KOK
Fitness						
Spring	0.93 (±1.07)	0.19 (±0.26)	0.58 (±0.70)	1.40 (±1.97)	0.42 (±0.59)	0.59 (±0.61)
Summer	0.18 (±0.33)	0.10 (±0.15)	0.01 (±0.02)	0.12 (±0.21)	0.14 (±0.35)	0.10 (±0.19)
Winter	0.05 (±0.07)	0.02 (±0.05)	0.02 (±0.02)	0.42 (±0.55)	0.11 (±0.14)	0.38 (±0.42)
Establishment	4314 (±2532)	119 (±83)	1263 (±814)	468 (±632)	170 (±270)	683 (±582)
Fertility	17 (±16)	148 ±205	162 (±183)	74 (±97)	8 (±6)	36 (±70)
Egg-L3						
Spring	0.14 (±0.14)	0.12 (±0.19)	0.35 (±0.52)	0.87 (±0.66)	0.88 (±0.77)	2.25 (±3.21)
Summer	0.05 (±0.02)	0.06 (±0.09)	0.01 (±0.02)	0.27 (±0.40)	0.38 (±0.62)	0.29 (±0.48)
Winter	0.01 (±0.02)	0.01 (±0.01)	0.01 (±0.01)	0.46 (±0.37)	0.31 (±0.34)	1.08 (±0.94)
Pathogenicity	19 (±6)	27 (±4)	24 (±2)	27 (±5)	30 (±1)	28 (±1)
FEC	11,299 (±10,166)	2940 (±5156)	4290 (±3581)	1765 (±2233)	257 (±395)	517 (±684)

Standard error of the mean (SEM).

## Data Availability

The data presented in this study are available on request.
